# Shielding a high‐sensitivity digital detector from electromagnetic interference

**DOI:** 10.1002/acm2.12366

**Published:** 2018-06-15

**Authors:** David E. Hintenlang, Xia Jiang, Kevin J. Little

**Affiliations:** ^1^ Department of Radiology Ohio State University Wexner Medical Center Columbus OH USA

**Keywords:** digital detector, electromagnetic, interference, mammography, shielding

## Abstract

**Purpose:**

To document a study in shielding a high‐sensitivity digital mammography system detector from AC magnetic fields of magnitudes great enough to induce imaging artifacts.

**Methods/materials:**

Preliminary evaluation of AC magnetic fields at a site designated for a digital breast tomosynthesis (DBT) system raised concerns that the magnetic component of electromagnetic interference (EMI) may be great enough to induce imaging artifacts. Subsequent measurements using digital detector arrays from two separate manufacturers verified this concern, and AC magnetic fields were mapped, spatially and temporally, throughout the area of concern. A simple shielding model was developed to elucidate the physics of extremely low‐frequency (ELF) EMI shielding and independently verify a commercial group's proposed shielding design and installation. Postshielding measurements were performed to demonstrate that the EMI fields were reduced to acceptable levels.

**Results:**

Preshielding measurements showed AC magnetic fields significantly exceeding manufacturers’ tolerances for artifact‐free imaging in DBT. Continuous measurements demonstrated that the EMI fields varied significantly over time. Some locations in the room routinely averaged above 30 mG and occasionally exceeded 100 mG. The source was attributed to an adjacent electrical supply room, and temporal changes of the EMI were attributed to variations of the building electrical loads. The proposed shielding primarily consisted of continuous aluminum (6.35 mm thickness) and was installed by a group specializing in electromagnetic field shielding. Postshielding measurements demonstrated that the EMI fields were significantly reduced, generally to less than 0.5 mG, and that the shielding effectively dampened the large variations due to dynamic building electrical loads. Subsequent installation and evaluation of a DBT system revealed no issues with imaging artifacts.

**Conclusions:**

The successful shielding of ELF EMI involves physical principles that are not commonly encountered by medical physicists. Modern high‐sensitivity digital detectors may be successfully shielded against imaging artifacts with careful application of these principles.

## 1 INTRODUCTION

Digital x‐ray detectors have evolved substantially over the past few years as manufacturers continue to make these devices increasingly sensitive. Multiple design strategies have been applied to digital imaging detectors, and modern advances provide high‐resolution arrays of small detector elements. In mammography, detectors have progressed toward incorporating direct or indirect detection schemes, both of which rely on an underlying array of thin‐film transistors (TFTs) to provide an electronic signal from each pixel element. Higher‐resolution imaging results in increasingly smaller elements and output signals, requiring substantial amplification as part of the readout process. This amplification may also provide opportunities for the inadvertent amplification of spurious noise or interference.

While not broadly recognized as an issue within the medical physics imaging community, manufacturers understand that high‐sensitivity detectors may be sensitive to a variety of environmental factors, including electromagnetic interference (EMI). Some of these effects may be well characterized in a laboratory environment but not necessarily in clinical environments. While some manufacturers provide specifications for the magnitude of electromagnetic (EM) fields that may be tolerated by their systems and recommendations for installation, these are not generally scrutinized during the selection and purchasing of new equipment. The site planning and pre‐installation guide for one manufacturer does not specify electromagnetic tolerances for the environment of use while that of another specifies power frequency (50/60 Hz) magnetic fields of less than 3 A m^−1^ (38 mG) per IEC 61000‐4‐8,^1^ although this was published prior to the latest generation of detectors becoming available. The latter manufacturer also includes a qualifying statement that these fields “should be at levels characteristic of a typical location in a typical commercial or hospital environment.”

This study documents a case study in evaluating and mitigating AC magnetic fields that were expected to produce imaging artifacts from EMI in a clinical mammography facility. While the production of EMI artifacts on images from digital detectors is becoming appreciated by manufacturers, there is little documentation in the literature that is directly applicable to clinical systems, although scientific presentations show examples of mammography and other digital detectors being affected under certain circumstances.^2^, ^3^, ^4^, ^5^ There is similarly a lack of literature for shielding clinical systems from AC magnetic fields at utility frequencies, which requires a substantially different approach from shielding the electromagnetic fields medical physicists most commonly encounter. MRI systems commonly require shielding of large, but static, magnetic fields and high‐frequency fields in the radiofrequency (RF) range. Static magnetic fields are best shielded by materials having high magnetic permeability, while high‐frequency fields may be readily shielded by thin sheets of highly conducting materials.^6^ The requirements for effectively attenuating frequencies employed for electrical utilities, 60 Hz in this case, fall between these two extremes.

This study presents reference measurements for 60 Hz AC magnetic field reduction strategies in a case where AC magnetic fields were found at magnitudes great enough to be of concern for artifact production for two manufacturers’ digital mammography systems. The reduction strategy demonstrates the effectiveness of shielding the examination room. Anecdotal discussions indicate that some previous attempts to perform EMI shielding under similar circumstances have met only limited success.

## 2 MATERIALS AND METHODS

Concern over the possibility of EMI‐generated artifacts on a proposed FFDM/DBT system installation incorporating a high‐sensitivity digital detector was initially raised by the mammography system manufacturer's representatives during the site selection process. These concerns were based on a recent experience at another unaffiliated facility. Initial AC magnetic field measurements made by the vendors’ representatives confirmed elevated AC magnetic fields of a magnitude great enough that image artifacts could be produced from interactions within the high‐sensitivity digital detector.

The vendor subsequently performed experimental testing on‐site with a test‐detector assembly. Results of this testing confirmed the presence of EMI‐induced artifacts and prompted a more thorough investigation. The possibility that the vendor's detector was uniquely sensitive to EMI prompted the solicitation of another vendor to also test their detector in this environment. The second vendor utilized a test detector based on an alternative detector design (indirect instead of direct detection). This system was similarly found to be sensitive to EMI artifacts at the levels of AC magnetic fields present in the planned mammography examination room. Neither test detector was connected to a full clinical system, but their data readouts indicated that an artifact would likely manifest itself as finely spaced alternating lines across the image.

### 2.A Source identification

An AC magnetic field survey by a vendor's representative was performed using a handheld gauss meter around the areas surrounding the proposed mammography examination room and readily revealed a source of concern. The examination room was located on the first floor of a multistory structure immediately adjacent to the main electrical supply room that included electrical distribution lines for the building. The main electrical loads for the building were distributed through this area and were expected to be responsible for the AC magnetic fields producing the EMI.

An obvious solution to reducing the potential interference was to relocate the examination room away from this focused source of electrical currents. Proposals to move the examination room to another location within the building were not considered viable due to administrative desires to maintain proximity to existing imaging clinic space, patient convenience, and other building occupancy constraints. A solution to shield the low‐frequency AC magnetic fields was selected to permit the digital mammography system to operate as intended in the selected space.

In order to more precisely determine the shielding design goals, additional measurements were made to characterize the EM fields of interest. Measurements were performed by both a commercial contractor and the institution's medical physics staff.

### 2.B Preshielding measurements

Measurements made prior to the design and installation of EM shielding were made by a commercial contractor (ETS‐Lindgren^1^ ) over the course of a single day. AC magnetic field measurements were performed for 60 Hz and the next two harmonics using a set of Bartington^2^ magnetometers. Measurements were performed along a volumetric grid throughout the proposed mammography exam room, with a total of 140 measurement positions. Measurement planes with heights of 0.305 m, 1 m, 1.52 m, and 2.43 m were used. Measurements were averaged over a period of 5 min and along three orthogonal axes for each measurement position.

Measurements performed by the in‐house medical physics team utilized a Reed^3^ Gu‐3001 magnetometer utilizing a uniaxial Hall effect sensor operated in AC mode. These measurements complemented the series performed by ETS‐Lindgren by providing additional survey information throughout the room, the ability to perform more detailed surveys of locally high readings and continuous measurements illustrating the temporal variation of fields at select locations in the area of interest. As well as providing independent confirmation of the area survey, measurements by the in‐house medical physics team focused on the possible time variation of the AC magnetic fields in the room due to varying electrical loads on the building. The magnetometer was interfaced to a laptop PC and acquired data continuously at 30 s intervals at the planned location of the mammography system detector and other locations of interest over multiple days. Continuous measurements were performed before and after the shielding installation process using this system.

### 2.C Mitigation plan

Based on the preliminary testing and the mammography system manufacturer's recommendations, a shielding installation was designed to reduce the AC magnetic fields to a level where imaging artifacts would not be expected to occur. Design goals were developed based on discussions with the mammography system manufacturer and on evaluations of AC magnetic field levels in some of our facilities with similar systems that do not exhibit any imaging artifacts. A design goal of 0.5 mG root mean square (rms) at the position of the mammography system detector was selected because it seemed to be a realistically achievable goal and was on the lower end of the range observed at other sites successfully operating without EMI artifacts. The shielding design, fabrication, and installation were outsourced to a commercial firm, ETS‐Lindgren.

An independent shielding model was developed by the medical physics team for a parallel review and to better understand the physics of electromagnetic shielding in the extremely low‐frequency (ELF, frequencies below 300 Hz) regime. The model is reviewed in the Discussion, along with generalized principles for designing ELF electromagnetic shielding that were largely devised by Schultz et al.^7^, ^8^, ^9^

The medical physics team provided consultation throughout the design process, and the design evolved through multiple iterations where differing amounts of wall coverage and material were proposed. The final shielding design specified the installation of 6.35 mm (0.25″) Al plate on all walls and the ceiling of the room, with the exception of two doors into the room which are located on the opposite side of the room from the electrical supply room. The floor was designed to have 6.35 mm (0.25″) Al plate over 6.35 mm (0.25″) M36 Silicon steel due to the presence of an under‐slab conduit that was found to run under the room. Additional details required that all joints and interfaces be covered by aluminum plates and/or stitch welded.

### 2.D Postshielding measurements

Following installation of the shielded structure, a series of measurements similar to the preshielding measurements were performed. This included a repeat of the test using a manufacturer's test‐detector assembly.

The commercial contractor (ETS‐Lindgren) performed a set of measurements using the same methodology employed for the initial characterization of the magnetic fields of interest. Measurements used the same detection process and a volumetric grid throughout the room at the same heights as the previous measurements.

The medical physics team similarly performed independent verification of these measurements, local surveys, and continuous measurements at select locations. Continuous measurements were made over multiple days in order to evaluate any effects that varying electrical loads of the building may have produced in the shielded room.

## 3 RESULTS

### 3.A Preshielding measurements

**Figure 1:**
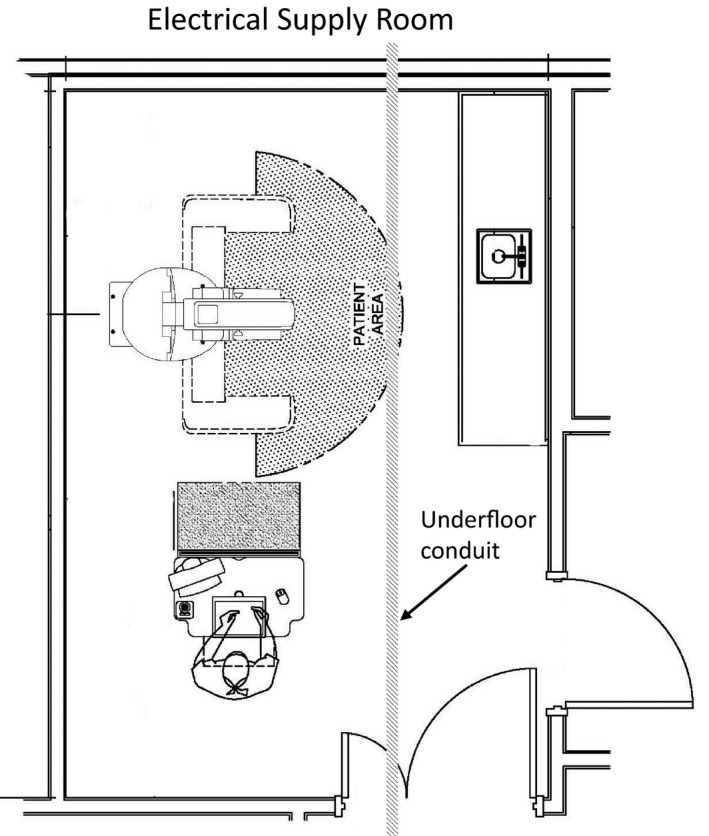
Plan view and location of relevant features associated with the mammography room.

**Figure 2:**
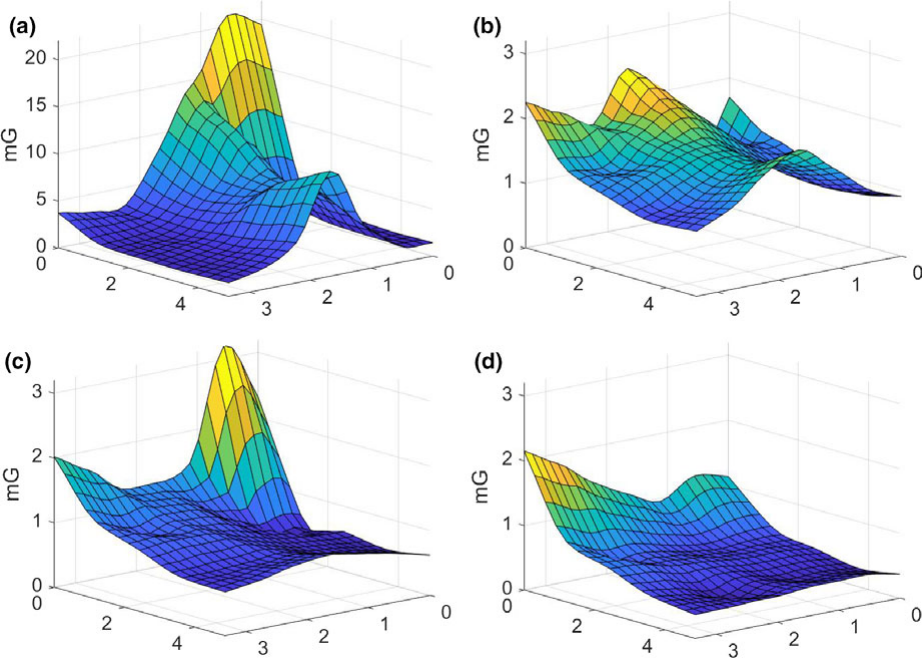
Surfaces illustrating the variation of the AC magnetic fields across the room at different elevations prior to shielding installation for horizontal planes located (a) 0.305 m, (b) 1 m, (c) 1.53 m, and (d) 2.44 m above the floor. Horizontal axes represent the room dimensions in meters. The wall bordering the electrical supply room is located at the back left, and the main door is located on the right front side of each diagram.

**Figure 3:**
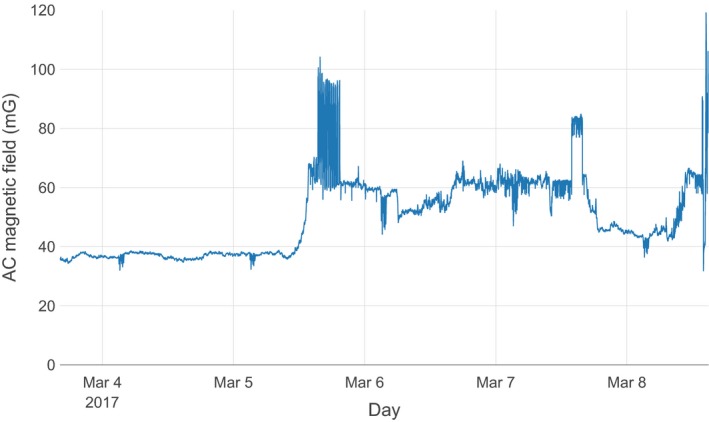
Temporal variations of the AC magnetic field prior to shielding installation at a point located 30 cm away from and at the midpoint of the wall bordering the electrical supply room and 30 cm above the floor. Midnight is delineated by the vertical gridline extending from each date.

**Figure 4:**
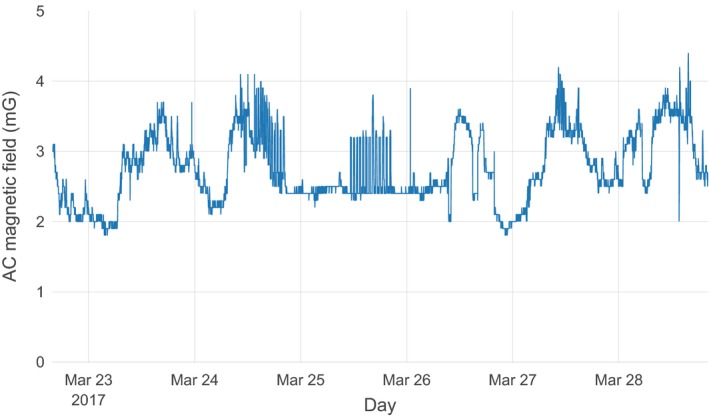
Temporal variations of the AC magnetic field prior to shielding installation at a point 1 m above the floor at the location of the digital detector. Midnight is delineated by the vertical gridline extending from each date.

Figure 1 illustrates the schematic layout of the room and the primary features of interest. Representative data from the array of measurements performed by the commercial contractor are illustrated in Fig. 2. The rms magnetic field from the three acquisition axes is illustrated by a surface in each figure. General trends observed were that the fields were usually the highest along the wall closest to the electrical supply room, initially decreased with distance from that wall, and then slightly increased near the opposite room wall. The figures also show that the intensity was greatest near the floor and decreased with increasing height. There were elevated areas of AC magnetic fields along the central axis of the room, extending from the center of the wall closest to the electrical supply room toward the doorway, and an electrical conduit was suspected to run along this path. Figures 3 and 4 show the temporal variation of the AC magnetic field at two different locations in the mammography room prior to the installation of EMI shielding. Data were acquired continuously at 30‐s intervals over an extended period of time. The measurement point for Fig. 3 was located 30 cm from the wall at the midpoint of the wall bordering the electrical supply room and 30 cm above the floor. This location consistently produced near the highest AC magnetic field measurements in the room. Data for Fig. 4 were collected at the proposed location of the mammography system detector and 1 m above the floor. Data were collected over several days at each location, and both figures illustrate that the AC magnetic field varied over the course of the day. General observations from this data suggest that there is less EM activity during weekends and that the magnitude of the activity can vary throughout the day, sometimes with very large and rapid fluctuations.

### 3.B Shielding installation

**Figure 5:**
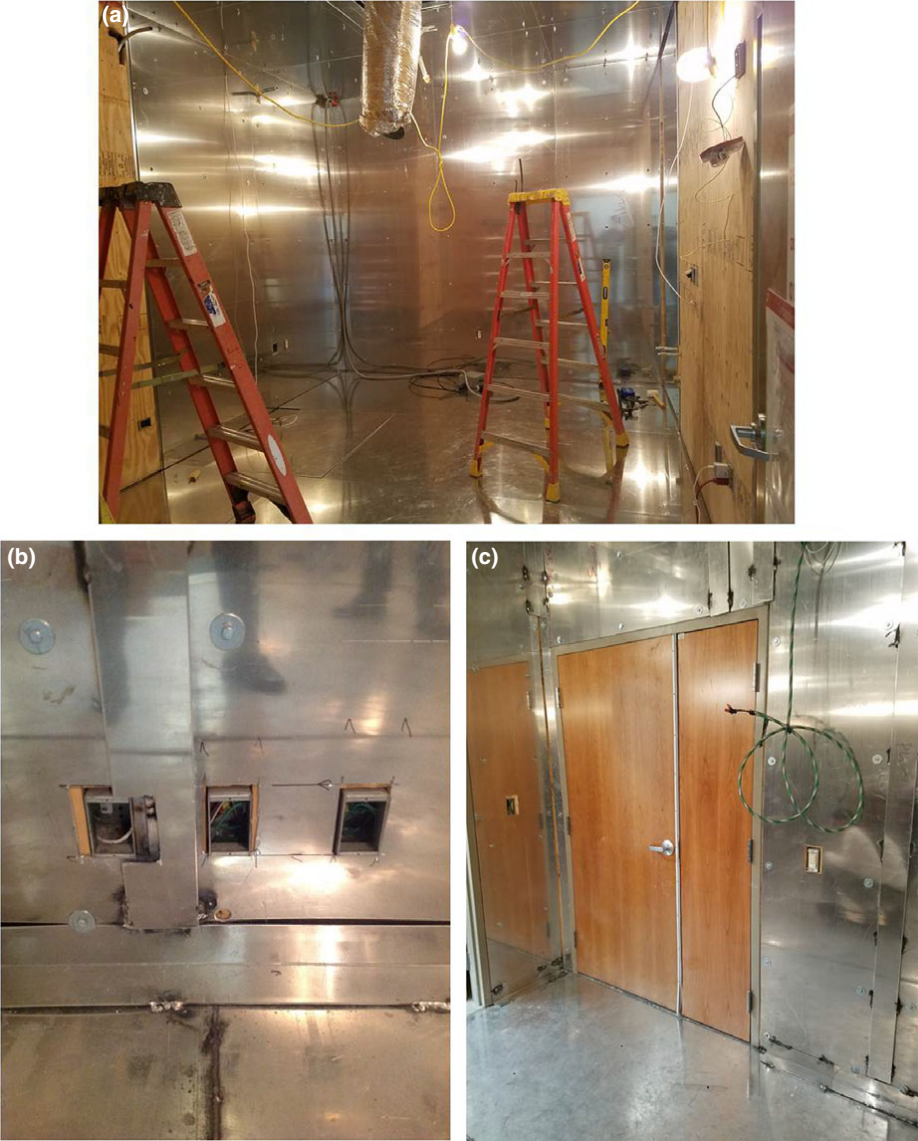
Aluminum shielding being installed. (a) Shielding installation complete on the far wall, floor, and ceiling. (b) Details of shielding around utility penetrations and illustration of the plates welded over Al sheet edges. (c) Al shielding installed around the main doorway which incorporates a wooden door.

The shielding was installed in accordance with the specified design over a period of approximately 2 weeks. After initial preparation, the 6.35‐mm (0.25″) Si‐steel plate was installed on the floor. Subsequently, 6.35‐mm (0.25″) thick aluminum was installed on the floor, walls, and ceiling, and minimal voids were cut in the aluminum for various utility penetrations. The wall installation utilized continuous aluminum sheets of 1.22 m (4 ft) width from floor to ceiling. The seams between adjacent sheets were stitch welded and covered with 6.35 mm (0.25″) aluminum strips to prevent EM leakage along the seams. Ceiling plates were hung from a superstructure that was installed for this specific purpose. Ceiling and floor plates were seam welded, and aluminum strips were installed over the seams. Of note, the doors to the room and to the adjacent changing area, located at the opposite end of the room from the electrical supply room, were traditional wood doors with no expected attenuation properties. Small penetrations were not further shielded since the maximum dimensions of the openings were much smaller than the wavelength of the EM waves and were not expected to permit significant EM leakage into the room. Figure 5 shows portions of the installation and the associated details.

### 3.C Postshielding measurements

**Figure 6:**
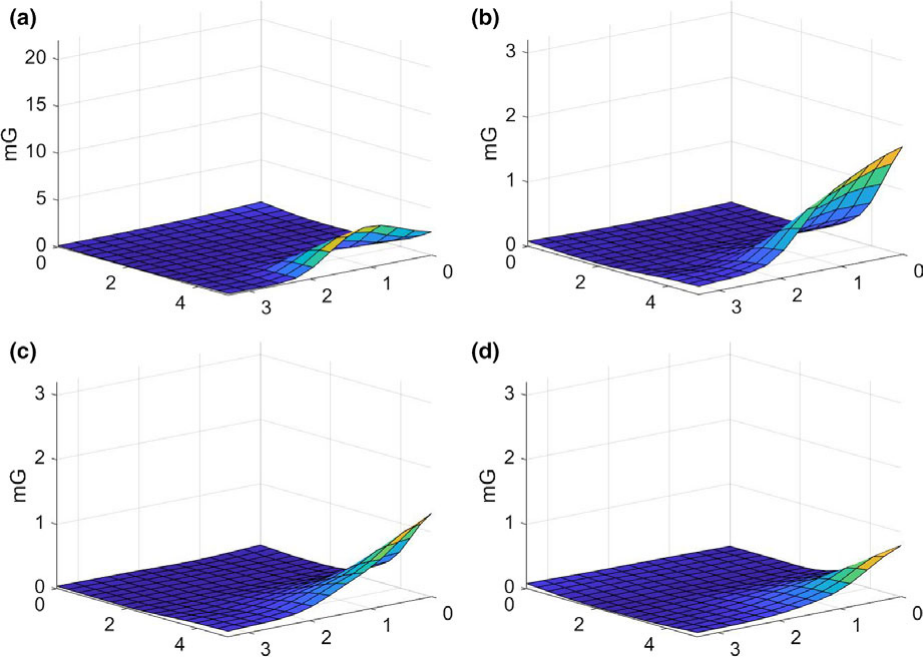
Surfaces illustrating the variation of the AC magnetic fields across the room at different elevations after shielding installation for horizontal planes located (a) 0.305 m, (b) 1 m, (c) 1.53 m, and (d) 2.44 m above the floor. Horizontal axes represent the room dimensions in meters. The wall bordering the electrical supply room is located at the back left, and the main door is located on the right front side of each diagram.

Representative data from the array of measurements performed by the commercial contractor following the completion of the EMI shielding installation are illustrated in Fig. 6. The rms magnetic field from the three acquisition axes is illustrated as a surface for each horizontal plane. General trends of these data demonstrate greatly reduced magnitudes of the AC magnetic fields compared with the preshielding measurements. The ridge of elevated magnitudes extending across the room due to an underfloor electrical conduit is no longer present. In all cases, the AC magnetic fields are quite low and uniform throughout most of the room but increase in magnitude near the unshielded doorways. Following this testing, the selected vendor again performed experimental testing on‐site with a test‐detector assembly. This testing revealed no signs of EMI interference on the detector and the finishing of the area, and installation was quickly completed.

**Figure 7:**
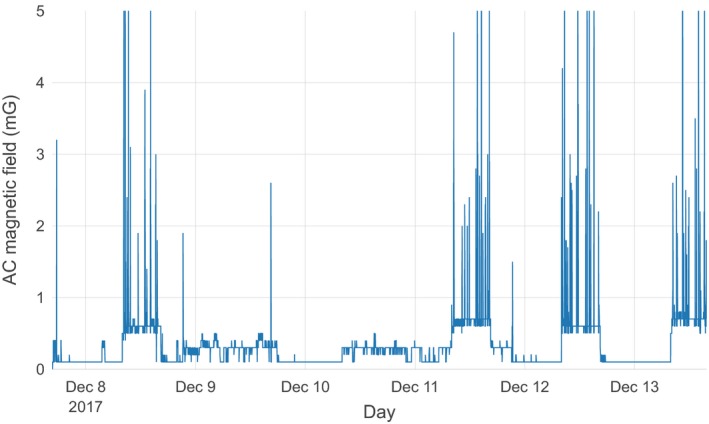
Temporal variations of the AC magnetic field after shielding installation at a point 1 m above the floor at the location of the digital detector. Midnight is delineated by the vertical gridline extending from each date.

Figure 7 shows the temporal variation of the AC magnetic field at the side of the installed gantry and 1 m above the floor following completion of the room. The continuous data illustrated in this figure were collected with the room completely finished and in operation. The EMI shielding was installed, all interior surfaces had been finished, and a Hologic Selenia Dimensions mammography system was installed and in clinical operation. The data exhibit a very different nature than the preshielding data. The average magnitude is much smaller and more constant. However, well‐defined periodic variations can be observed, and discrete spikes occur throughout the day. No image artifacts that could be attributed to EMI have been observed during the 3 months that the system has been in clinical use.

## 4 DISCUSSION

### 4.A Preshielding measurements

Measurements that were performed prior to the design and installation of the shielding were useful in determining the magnitude of the AC magnetic fields present in the room and identified potential EMI sources. The magnitudes correlated with the expectation that the primary source was the electrical supply room located adjacent to the mammography room since the magnitudes were greatest along this wall and decreased with distance away from it. An additional source was an electrical supply conduit that ran from this wall under the room toward the doorway and that was associated with the ridge that is observed on the surface plots in Fig. 2. The magnitude of the AC fields also decreased with distance away from the floor.

Two mammography system manufacturers tested high‐sensitivity digital detectors in the room, and both observed EMI anomalies during the data readout. Concerns were that this could lead to streaks, or lines, across the image. Neither manufacturer would definitively state whether these anomalies would lead to, or would not lead to, artifacts in clinical images for either the pre‐ or postshielding magnetic field environment. Following discussions with the manufacturers’ engineers, a design criteria of 0.5 mG was selected as the design goal for the 60‐Hz magnetic fields in the room, specifically at the detector position. Even at this level, neither of the manufacturers would guarantee that EMI artifacts would not appear in clinical images.

Figures 3 and 4 demonstrate that the magnitude of magnetic fields also varied significantly over time. There were clear variations that took place over the course of each day, and different behaviors were also observed on weekends versus business days. Figure 3, which was measured in close proximity to the floor and electrical supply room wall, shows an average of around 36 mG and relatively constant magnetic fields during a measurement period extending over a Saturday and a Sunday. A rapid increase to around 60 mG occurred around noon on Monday, with a number of rapid variations extending to greater than 90 mG throughout the day. The magnetic fields remained elevated with excursions both above and below 60 mG over the next several business days. Figure 4 illustrates the temporal variation at the proposed detector location. The AC fields at this location had smaller peak magnitudes and were more diurnally periodic in nature, varying from around 2.0 to 4.0 mG during weekdays. During the weekend days, the diurnal variation was not observed, with a nearly constant baseline of 2.5 mG (although several periods of rapid variation occurred on Saturday, March 25). These variations are attributed to the varying electrical loads from the operation of building equipment and systems throughout the day.

### 4.B Shielding design and installation

**Figure 8:**
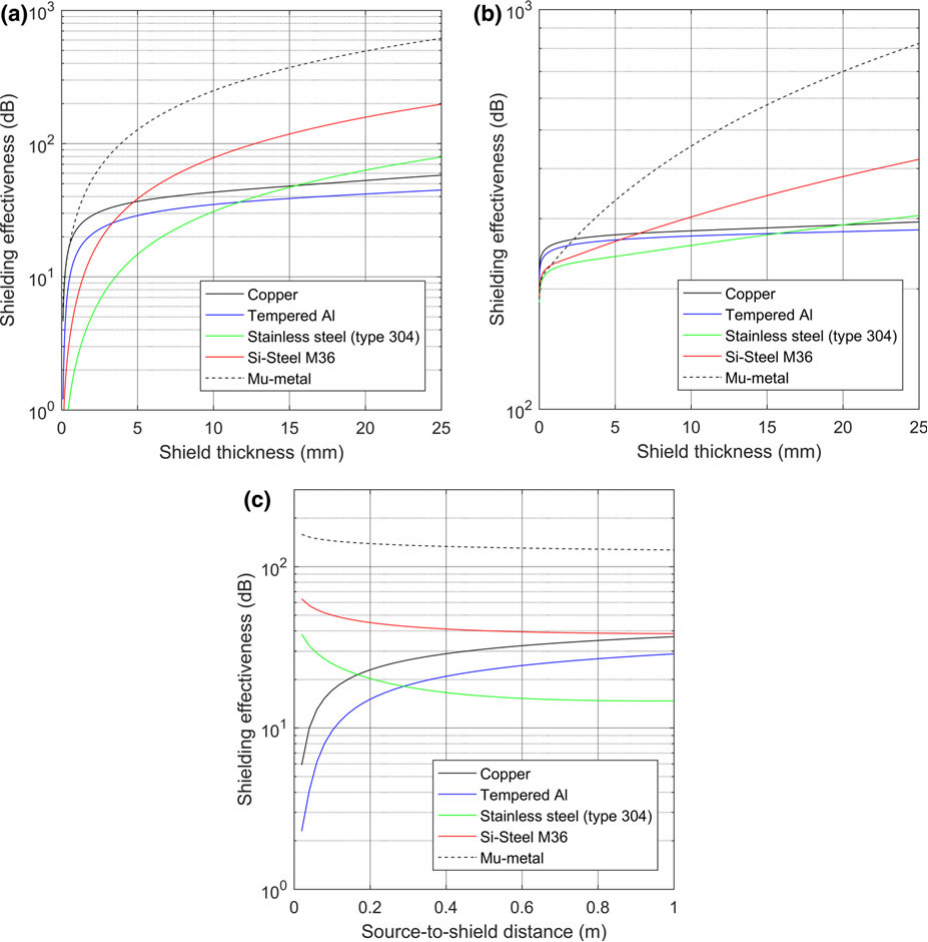
Shielding effectiveness of (a) the magnetic field and (b) the electric field as a function of shield thickness for common shielding materials at an assumed source‐to‐shield distance of 1 m. (c) Shielding effectiveness of the magnetic field as a function of source‐to‐shield distance for 5‐mm‐thick attenuating materials.

The shielding design progressed through a number of planning discussions and several iterations before the final design was achieved. Initial discussions focused on an appropriate shielding material for the effective reduction of AC magnetic fields with frequencies around 60 Hz. While the medical physics team expected that a material of high magnetic permeability would be most appropriate for shielding magnetic fields, the initial design proposed by the commercial shielding design group, which was based on their proprietary electromagnetic modeling, primarily used aluminum sheet. In order to better understand the rationale for this selection, a simple model was adapted to estimate shielding effectiveness using an infinite plate model and matching Maxwell's equations to the appropriate boundary conditions.^7^, ^8^, ^9^, ^10^ While coupled electric and magnetic fields are present, it is expected that the EMI fields originate from low‐impedance sources where the magnetic field dominates in the near field.^11^ In principle, it is necessary to attenuate both the coupled electric and magnetic field components of the EM field. Predictions of shielding effectiveness (attenuation expressed in decibels) for both components were developed from previously published nomograms^12^ and are illustrated in Fig. 8 for 60‐Hz fields. Figure 8 demonstrates that the smaller electric field component is readily attenuated by conductive materials and that common steel materials are more effective in attenuating the magnetic field component than aluminum when large amounts of attenuation are required or the shielding is close to the source. As illustrated in Fig 8(a), at a source‐to‐shield distance of 1 m, reasonable thicknesses of aluminum can provide attenuation of the magnetic field component up to around 40 dB and even shield more effectively than Si‐Steel for shield thicknesses less than 3.5 mm. At shorter source‐to‐shield distances, the shielding effectiveness of aluminum decreases relative to that of Si‐steel, as illustrated in Fig. 8(c).

Ultimately, considerations beyond shielding effectiveness that included weight, workability, and cost factored into the decision to utilize aluminum as the predominant shielding material. The final shielding design was based on utilizing sheets of 6.35 mm (0.25″) aluminum on the walls and ceiling and 6.35 mm (0.25″) aluminum overlying 6.35 mm (0.25″) M36 Si‐steel on the floor to achieve a greater level of attenuation from the sources located a short distance below the floor.

The geometry of an earlier proposed design provided complete shielding on the floor, the wall bordering the electrical supply room, and the ceiling but only a portion of two other walls. This raised concerns that the low‐frequency EMI would bypass the installed shielding and continue to affect the mammography system. This design was rejected and revised to run aluminum from ceiling to floor for all walls. Thus, all interior room surfaces were covered with aluminum with the exception of the two doorways, which were not specified to incorporate any attenuating materials and utilized the existing wooden doors. It was considered that they could later be replaced with steel doors if necessary.

### 4.C Postshielding evaluation

As previously described, a series of detailed measurements were made following the completion of the shielding installation. A direct comparison of the pre‐ and postshielding measurements performed throughout the room volume (Figs. 2 and 6) demonstrates that the shielding provided significant attenuation of the AC magnetic fields throughout the room. Areas of large magnitude fields from both the electrical supply room and the underfloor electrical conduit were heavily attenuated and indistinguishable from other low‐field areas of the room. The practical effectiveness of the shielding design can be illustrated by comparing pre‐ and postshielding AC magnetic fields around the location of the system's digital detector. Before shielding, the average of measurements around the detector location was 1.6 mG, and afterward the average was 0.04 mG, which was below the design goal of 0.5 mG.

The postinstallation measurements all demonstrated that the highest remaining AC magnetic field strengths occur in the region of the room where the two unshielded doorways were located. Prior to the shielding installation, these locations were among the lowest measured within the room. After the shielding installation, these locations exhibited very little change in the magnitude of the magnetic fields, becoming the highest magnitude areas in the room. This was attributed to EM leakage into the area from the electrical conduit that ran along the length of the room near the main doorway and perhaps leakage of other EM sources into the area. This highlights the importance of completely shielding an area in order to reduce EMI intrusion. Fortunately, the mammography system detector was located far enough away from the doorway that the EM fields were reduced below the design goal. A successful outcome would likely not have been achieved if the early design proposal that only provided partial shielding of two walls had been adopted.

Continuous measurements were also made in the room after the shielding was installed and the room was finished. Due to construction activity, measurements were not attempted until after the system was installed and in clinical use. Temporal measurements for a period covering 6 days under clinical operation are illustrated in Fig. 7. These measurements were consistent with an overall reduction in the AC magnetic field activity, with baseline magnitudes less than 0.1 mG, but large peaks were also observed. As with the preshielding measurements, AC magnetic field activity was quite low on weekend days but increased with weekday activity. Upon a closer examination the increased weekday activity was observed to coincide precisely with the hours of operation of the mammography system, 8:30 am to 4:00 pm. Furthermore, the large spikes in magnetic fields were observed to coincide with mammography acquisition times. A subsequent series of measurements verified that the large spikes occurred during mammography exposures in both conventional 2D and tomographic modes. The greatest magnitude was observed in close proximity to the x‐ray tube but remained measureable in many areas of the room.

Evaluation of both quality assurance and clinical images collected on the mammography system did not demonstrate any EMI‐related artifacts. Close examination of flat‐field images showed that the images were representative of flat‐field images collected on a variety of similar systems. We concluded that the installed shielding successfully reduced the building‐produced EM fields to levels that were not observable on this digital detector system as interference and that the system was not responsive to the fields measured during the x‐ray exposure. This was consistent with EM fields affecting the detector readout process, which would occur following the exposure.

Anecdotal information from another facility that installed EMI shielding on a single surface between the expected EM source and the mammography detector system suggested that the shielding at that facility was less successful in preventing imaging artifacts. As indicated by the continued presence of EM fields in the areas around the unshielded doorways, a robust approach must be taken to shield all areas surrounding sensitive digital systems to be assured that the magnetic fields are reduced to levels that will not produce imaging artifacts.

As a result of the expenses and delays in completing a project where EMI may be encountered, we now perform AC magnetic field surveys at all locations that are considered for digital mammography system installations. With our institution's current trend toward locating mammography installations at a variety of off‐site locations, many of which may be leased, AC magnetic field surveys are performed at all candidate locations prior to committing to site a facility. Since the construction details of a leased facility may not be well known by the institution, an on‐site survey can identify problematic areas for siting a digital mammography system. At our institution, these measurements are performed by both the in‐house medical physics staff and the manufacturer's representative at different portions of the site selection process. Most areas in institutional buildings surveyed have been observed to have fields in the range of 0.3–0.6 mG, which have not produced any imaging artifacts. We believe the surveys have been useful, and in one case, we identified an area that would likely have been problematic because the electric distribution panel for an array of automotive recharging stations was located near the proposed mammography location. Anecdotal discussions suggest that our experience with EMI artifacts and the need to relocate or shield mammography systems utilizing high‐sensitivity detectors is not unique. As the sensitivity and performance of digital detectors continues to improve, it will likely be increasingly important to characterize the electromagnetic environment where they will be located to minimize the possibility of environmentally induced imaging artifacts.

## 5 CONCLUSIONS

High‐sensitivity digital detectors incorporated into modern mammography systems have been observed to be more susceptible to electromagnetic interference from AC magnetic fields than previous generations of detectors. This may extend to digital detectors integrated into other clinical applications as well and strongly suggests that the clinical environment where sensitive detectors will be deployed should be evaluated for potential image artifact‐producing EMI. Presiting surveys provide an effective tool for identifying potential problem areas early in the process. If problems are identified, the most effective option is usually to consider a different location or site. If other options are not available, we have demonstrated that it is possible to successfully shield a system from AC magnetic field interference in order to avoid imaging artifacts.

The physics of shielding AC magnetic fields fundamentally differs from the shielding of other electromagnetic sources or ionizing radiation commonly encountered by a diagnostic medical physicist in the clinic. Unlike the strategy used to attenuate ionizing radiation where an attenuator is placed only between the source and area of interest, EMI shielding should be expected to totally enclose the area of interest. In contrast to the extremes of static magnetic field shielding and high‐frequency RF field shielding necessary in MRI, we found that a combination of modest magnetic permeability and good conductivity materials can provide good attenuation of magnetic fields in the range of 60 Hz. High magnetic permeability is a desirable property of shielding materials, but in cases where only a modest amount of attenuation is required, lower permeability materials with good electrical conductivity can be quite effective. In this case, a combination of aluminum and steel was used for EMI shielding, providing an easier and less expensive installation than more exotic materials.

## CONFLICT OF INTEREST

No conflicts of interest.

## NOTES

1 ETS‐Lindgren Inc., 1360 N Wood Dale Rd, Suite G Wood Dale, IL 60191

2 Bartington Instruments Ltd., Thorney Leys Business Park, Witney, Oxfordshire OX28 4GE, UK

3 Reed Instruments, http://www.reedinstruments.com
